# Ku Stabilizes Replication Forks in the Absence of Brc1

**DOI:** 10.1371/journal.pone.0126598

**Published:** 2015-05-12

**Authors:** Arancha Sánchez, Paul Russell

**Affiliations:** Department of Cell and Molecular Biology, The Scripps Research Institute, La Jolla, California, United States of America; RIKEN Advanced Science Institute, JAPAN

## Abstract

DNA replication errors are a major source of genome instability in all organisms. In the fission yeast *Schizosaccharomyces pombe*, the DNA damage response protein Brc1 binds phospho-histone H2A (γH2A)-marked chromatin during S-phase, but how Brc1 protects genome integrity remains unclear. Here we report that the non-homologous end-joining (NHEJ) protein Ku becomes critical for survival of replication stress in brc1∆ cells. Ku’s protective activity in *brc1∆* cells does not involve its canonical NHEJ function or its roles in protecting telomeres or shielding DNA ends from Exo1 exonuclease. In *brc1∆ pku80∆* cells, nuclear foci of Rad52 homologous recombination (HR) protein increase and Mus81-Eme1 Holliday junction resolvase becomes critical, indicating increased replication fork instability. Ku’s localization at a ribosomal DNA replication fork barrier associated with frequent replisome-transcriptosome collisions increases in *brc1∆* cells and increased collisions correlate with an enhanced requirement for Brc1. These data indicate that Ku stabilizes replication forks in the absence of Brc1.

## Introduction

Genome integrity is especially vulnerable during the DNA synthesis (S) phase of the cell cycle, when replisomes encounter DNA lesions, DNA-bound proteins and opposing transcriptosomes. Limiting supplies of deoxyribonucleotides and oncogene activation also cause replicative stress. Genome maintenance proteins insure accurate genome duplication during replicative stress, with the paramount factor being the master checkpoint protein kinase known as ATR in humans, Mec1 in the budding yeast *Saccharomyces cerevisiae* and Rad3 in the fission yeast *Schizosaccharomyces pombe* [1–4]. The carboxyl terminus of histone H2A in yeasts and H2AX in mammals is a key substrate of these checkpoint kinases [5]. Phospho-H2A/X (γH2A/X) is best known for its functions at double-strand breaks (DSBs) but it also marks a diverse array of genomic features during S-phase in fission yeast, including natural replication fork barriers, retrotransposons, heterochromatin in the centromeres and telomeres, and ribosomal RNA (rDNA) repeats [6]. Indeed, γH2A is required for full resistance to genotoxins that cause replicative stress [7]. A key role of γH2A in S-phase was revealed by the discovery that Brc1 genome protection protein forms nuclear foci by binding γH2A during replicative stress [8, 9]. X-ray crystallography of a Brc1-γH2A peptide complex showed that the C-terminal region of Brc1 consisting of tandem BRCT domains folds to form a highly sculpted docking pocket for the phospho-SQE motif at the carboxyl-tail of γH2A. Missense mutations in this docking pocket ablate Brc1 foci formation and confer sensitivity to replicative stress [8].

Despite these insights it remains unclear how Brc1 actually protects genome integrity during replicative stress. Brc1 was first identified as high copy suppressor of the hypomorphic *smc6-74* mutation, which compromises the activity of the Smc5-Smc6 holocomplex that is essential for structural maintenance of chromosomes and has important but poorly understood roles in DNA repair [10–12]. Brc1 is not required for cellular viability but it is essential in strains with defective functions of the Smc5-Smc6 complex [11, 13, 14]. Brc1-null strains are sensitive to DNA damaging agents and drugs that cause replication fork arrest or collapse [9], and the appearance of Rad52 foci in untreated *brc1∆* cells suggest DNA replication difficulties even in the absence of exogenous genotoxins [8, 15]. Brc1-coated chromatin might promote the bypass of DNA lesions by post-replication repair (PRR) or it might stabilize stalled replisomes [6, 15, 16]. Unstable replication forks are prone to collapse and breakage, necessitating engagement of the homologous recombination (HR) repair machinery to reestablish the replication fork [17, 18]. Indeed, the repair of a site-specific broken replication fork in fission yeast absolutely depends on key HR proteins such as Rad51 and Rad52 recombinases and Mus81-Eme1 Holliday junction resolvase, but not on other DSB repair factors such as Ku that are required for non-homologous end joining (NHEJ) [19]. The absence of an acute requirement for Rad51 or Mus81 in *brc1∆* cells suggests that replication fork collapse does not increase in *brc1∆* mutants [9], even though these cells have more Rad52 foci [8, 15].

Here, we report on our latest efforts to determine how Brc1 protects genome integrity during S-phase. One of our key findings is that elimination of Ku in *brc1∆* cells reveals a critical requirement for Mus81, which indicates that Ku stabilizes replication forks in the absence of Brc1. Our studies shed new light on a non-canonical function of Ku and the role of Brc1 in protecting cells from replicative stress.

## Materials and Methods

### Strains and genetic methods

The strains used in this study are listed in S1 Table. Standard fission yeast methods were used as described previously [20]. Deletion mutations strains were constructed as described previously [21]. Successful deletion of these genes was verified by PCR. Tetrad analysis was performed to construct double mutants and verified by PCR.

The E-MAP screen methods and workflow were performed and normalized as previously described [22]. Genetic interaction score were determined with a simple growth phenotype that measures negative interactions, such as synthetic sick/lethal (SSL) interactions (E-MAP score < -2.5), as well as positive interactions (E-MAP score > 2) in which the double mutant is healthier than would be expected based on the growth of the two single mutants.

For synchronization of cells using *cdc25-22* block and release, cells containing the temperature sensitive *cdc25-22* allele were incubated at restrictive temperature (36°C) for 4 hours to arrest the cell cycle in G2-phase. Upon release to permissive temperature (25°C) the cells synchronously enter the cell cycle. Cell cycle progression was monitored microscopically by counting cells that contained septa, the appearance of which correlates with S-phase.

### Survival assay

DNA damage sensitivity assays were performed by spotting 10-fold serial dilutions of exponentially growing cells onto yeast extract with glucose and supplements (YES) plates, and treated with indicated amounts of hydroxyurea (HU), camptothecin (CPT), and methyl methanesulfonate (MMS). For UV treatment, cells were serially diluted onto YES plates and irradiated using a Stratagene Stratalinker UV source. Cell survival was determined after 3–4 days at 30°C.

### Telomere analysis

Genomic DNA isolated from each strain was digested overnight with EcoRI and resolved in 2% TAE agarose gel. DNA was transferred via capillary method to an Amersham Hybond-XL membrane (GE Healthcare Life Sciences) and probed with ^32^P-labeled TAS1 [23].

### Microscopy

Cells were photographed using a Nikon Eclipse E800 microscope equipped with a Photometrics Quantix charge-coupled device (CCD) camera and IPlab Spectrum software. All fusion proteins were expressed at their own genomic locus. Rad52-yellow fluorescence protein (YFP) and RPA(Rad11)-green fluorescence protein (GFP) expressing strains were grown in EMM media until mid-log phase for focus quantification assays. 500 or more nuclei were scored in three independent experiments.

### Chromatin immunoprecipitation (ChIP) assay

Real time qPCR ChIP experiments were performed as described previously [6, 24] using anti-HA antibody (Roche Applied Science) conjugated to anti-mouse magnetic beads (Invitrogen) to precipitate Pku70-HA expressed at wild type levels from the endogenous locus under the control of the native *pku70* ^+^ promoter. qPCR primers are from [6]. Percent of immunoprecipitation DNA (%IP) in the ChIP samples was calculated relative to the amount of DNA in the input samples. ChIP enrichment was calculated relative to *act1*. All error bars represent the standard error between technical triplicates.

## Results

### Requirement for Ku in *brc1∆* mutant

To gain new functional insights into Brc1 we generated an epistatic miniarray profile (E-MAP) consisting of the quantitative analysis of genetic interactions between *brc1∆* and a *S*. *pombe* gene deletion library of ~2,200 nonessential genes [25–27]. A full description of this E-MAP is in preparation; here, we focus on the synthetic sick/lethal (SSL) genetic interaction with Ku. The *brc1∆ pku80∆* double mutant generated an E-MAP score of -5.4, which ranks 19^th^ amongst the 2,027 E-MAP scores obtained in this screen in which an E-MAP score below -2.5 was judged to be significant. For comparison the previously analyzed E-MAP interaction with the deoxycytidylate deaminase *dcd1∆* mutation, which causes a deoxyribonucleotide imbalance that creates replicative stress, were -7.8 and -7.6 for the two *dcd1∆* mutants tested in the library [25].

**Fig 1:**
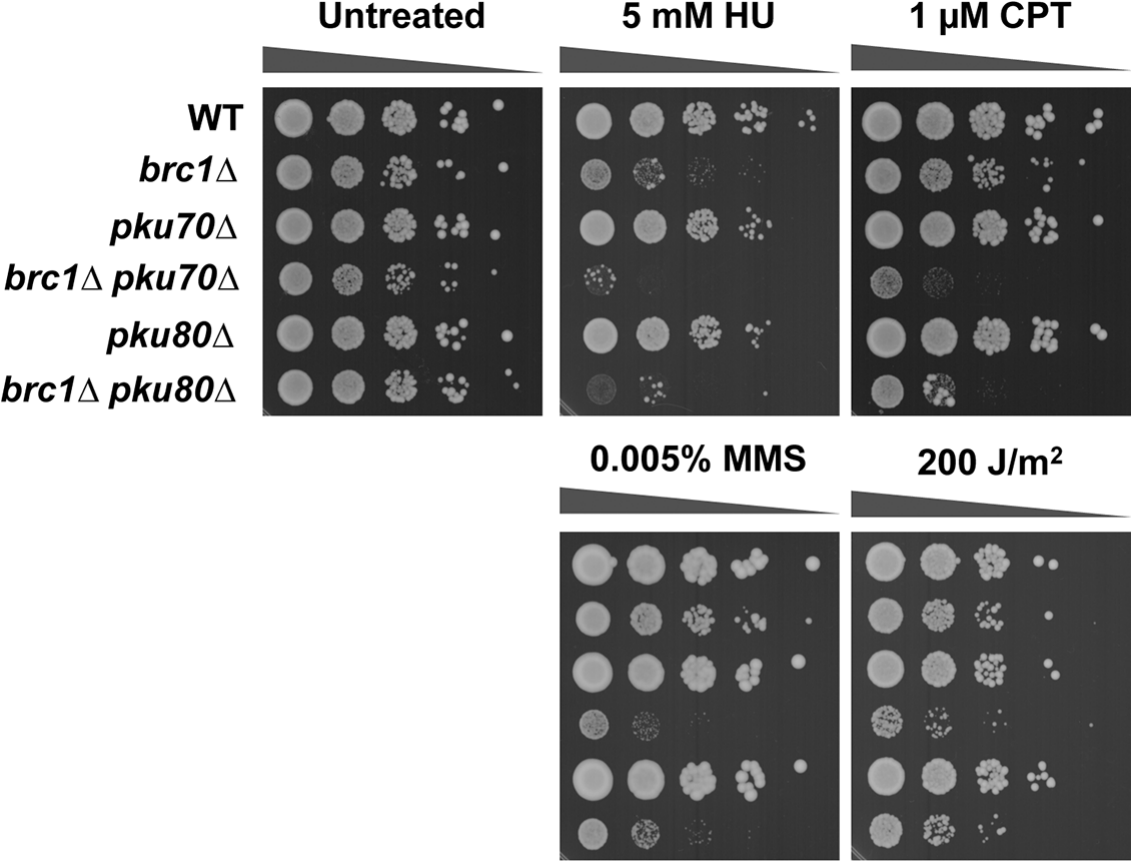
DNA damaging agents enhance the requirement for Ku in *brc1∆* cells. Tenfold serial dilutions of cells were exposed to the indicated DNA damaging agents. Plates were incubated at 30°C for 3 or 4 days.

Comparisons made with a panel of genotoxins that cause replicative stress showed that a *brc1∆ pku80∆* double mutant was much more sensitive than either single mutant (Fig 1). This effect was observed with hydroxyurea (HU), which slows replication by inhibiting ribonucleotide reductase, and with agents such as UV light, camptothecin (CPT) and methyl methanesulfonate (MMS), which create several types of DNA lesions that cause replication forks to stall or collapse. Note that these serial dilution assays indicated only a weak SSL interaction in the *brc1∆ pku80∆* double mutant grown in the absence of genotoxins, which attests to the sensitivity of the E-MAP assay (Fig 1).

The *pku70∆* mutation was absent from the version of the haploid deletion library used in our screen. We therefore generated *brc1∆ pku70∆* cells to specifically test whether the Ku heterodimer is important in the absence of Brc1. As predicted, these cells displayed an SSL interaction that was accentuated in the presence of the aforementioned genotoxins (Fig 1).

### Non-canonical requirement for Ku in the absence of Brc1

Ku consists of the Ku70-Ku80 heterodimer that forms a ring that encircles duplex DNA by sliding onto the DNA end. Ku binds DSBs with very high affinity, whereupon it links DNA ends to initiate NHEJ repair [28]. NHEJ is not generally known to be involved in the repair of replication-associated DNA damage, thus its genetic interaction with Brc1 was unexpected. As Ku is absolutely essential for NHEJ, whereas Brc1 is one of many pathways that prevent replication-associated DNA damage or participate in its repair, we suspected that the Brc1-Ku SSL interaction indicated a role for Ku in recovery from replicative stress. Indeed, an SSL interaction between Ku and Rqh1 DNA helicase, which is involved in multiple DNA repair pathways and DNA replication but not NHEJ, suggested that Ku has an unappreciated role in genome protection during S-phase [29].

**Fig 2:**
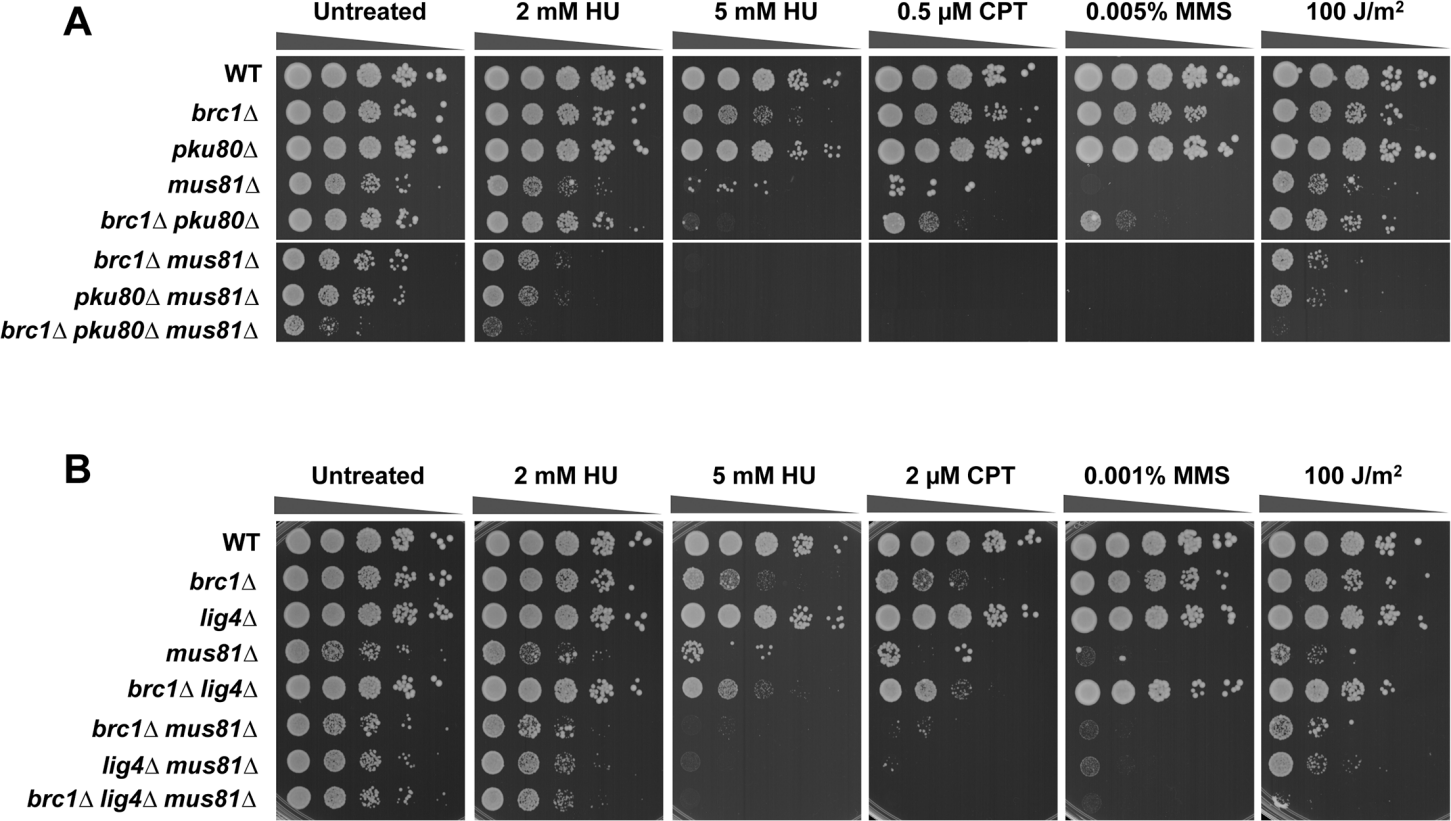
Mus81 Holliday junction resolvase is critical in *brc1∆ pku80∆* cells. Genetic interactions amongst Brc1, Mus81 and Pku80 (A) or Lig4 (B). Tenfold serial dilutions of cells were exposed to the indicated DNA damaging agents. Plates were incubated at 30°C for 3 to 4 days.

To formally examine whether the NHEJ defect of *pku80∆* mutants is responsible for the SSL interaction with *brc1∆*, we tested whether DNA ligase IV, which is essential for the final ligation step of NHEJ, was required in *brc1∆* cells. No SSL interaction was evident in *brc1∆ lig4∆* cells (Fig 2B). Furthermore, we found that *lig4∆* did not enhance genotoxin sensitivity in *brc1∆* cells, unlike the effect of *pku80∆* in *brc1∆* cells. These findings establish that the requirement for Ku in *brc1∆* cells does not involve NHEJ.

**Fig 3:**
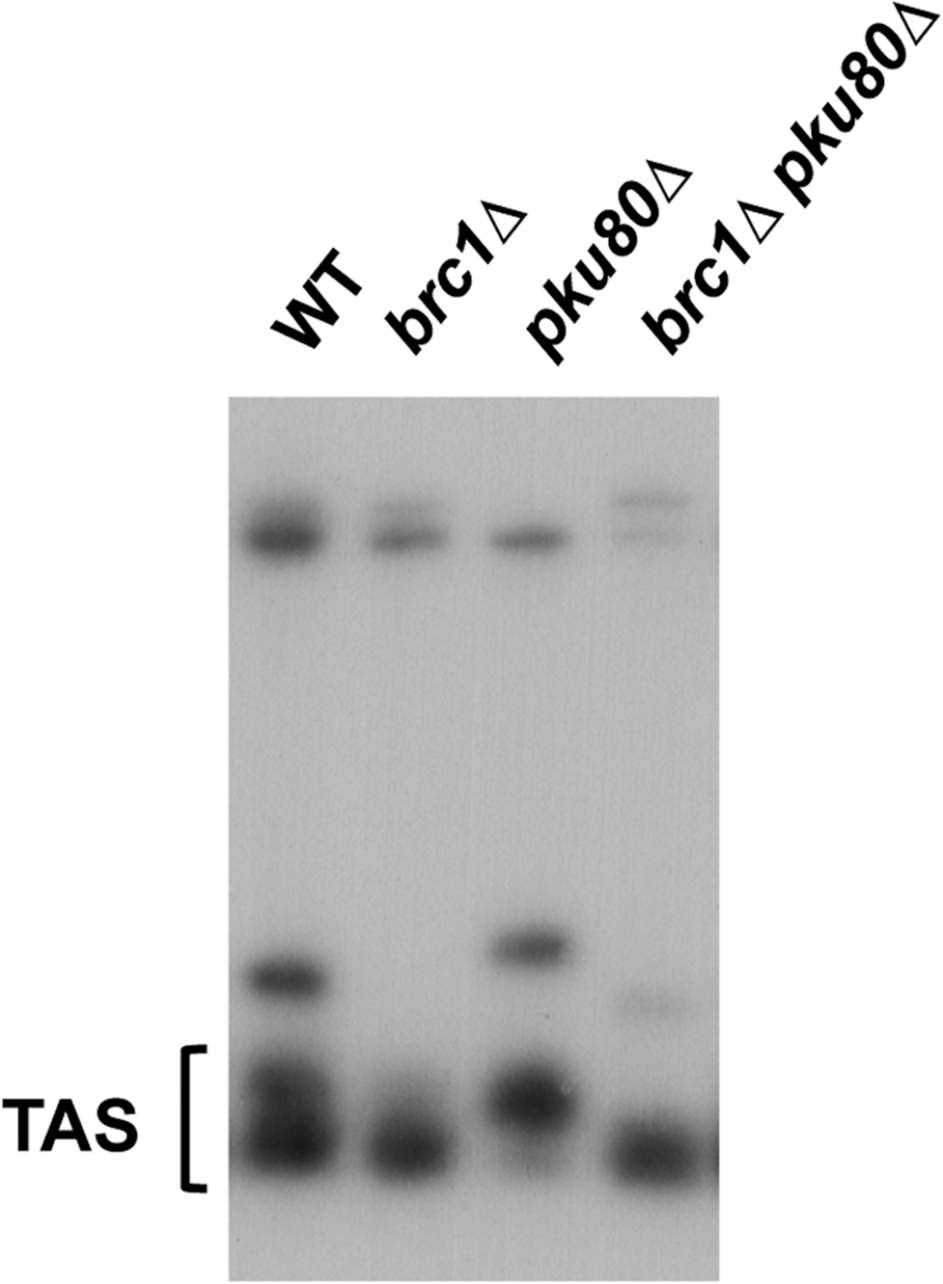
A *brc1∆ pku80∆* strain maintains telomeres. Southern blot analysis of EcoRI-digested genomic DNA from the indicated strains probed with telomere associated-1 (TAS1) DNA.

Ku participates in telomere maintenance in fission yeast [30]. Rearrangements of telomere-associated sequences (TAS) were reported to increase during meiosis and/or germination in *pku80∆* cells [31]. To assess whether the SSL interaction between *brc1∆* and *pku80∆* might involve catastrophic loss of TAS sequences, we performed a cross of *brc1∆* and *pku80∆* and analyzed TAS by Southern blotting. Our data indicated that the *brc1∆ pku80∆* double mutant retained TAS sequences in a pattern that was quite similar to the *brc1∆* parent (Fig 3). Thus, the SSL interaction between *brc1∆* and *pku80∆* does not appear to involve defects in telomere maintenance.

### Critical requirement for Mus81-Eme1 in *brc1∆ pku80∆* cells

The SSL phenotypes of *brc1∆ pku80∆* cells suggested that they might suffer increased replication fork collapse or rearrangement in response to replication stress. Repair of these events typically requires resolution of Holliday junctions by Mus81-Eme1 endonuclease, which is the sole nuclear Holliday Junction resolvase in fission yeast. This requirement explains why *mus81∆* mutants are acutely sensitive to genotoxins that cause replication fork collapse but are insensitive to ionizing radiation and other types of clastogens that create DSBs independently of DNA replication [32–34]. To test whether the SSL interaction involving Brc1 and Ku involves replication fork instability, we analyzed their genetic interactions with Mus81. In untreated conditions, *brc1∆ mus81∆* and *pku80∆ mus81∆* cells displayed nearly the same growth defect as *mus81∆* cells, confirming that loss of neither Brc1 nor Ku leads to a large increase in replication fork collapse. In contrast, the triple mutant *brc1∆ pku80∆ mus81∆* displayed a severe growth defect (Fig 2A, untreated). These data strongly suggested that SSL interaction involving *brc1∆* and *pku80∆* results in replication fork instability leading to the formation of HJs or related DNA structures that must be resolved by Mus81-Eme1. This interaction was apparent in the absence of genotoxins, indicating that endogenous conditions that impede replication are more likely to result in replication fork collapse or rearrangement in *brc1∆ pku80∆* cells.

Interestingly, both *brc1∆ mus81∆* and *pku80∆ mus81∆* strains displayed enhanced sensitivity to HU and UV as compared to the relevant single mutants (Fig 2A). The genetic interaction between *pku80∆* and *mus81∆* is particularly interesting because it indicates that *pku80∆* mutants suffer increased replication fork instability in the presence of these genotoxins. Note that potential synergistic effects in CPT and MMS could not be assessed in this experiment because of the acute sensitivity of the *mus81∆* mutant to these genotoxins (Fig 2A).

In contrast to *pku80∆*, the *lig4∆* mutation did not impair growth in the *brc1∆ mus81∆* background, which further supports the conclusion that the requirement for Ku in *brc1∆* does not involve its function in NHEJ (Fig 2B). The absence of genetic interactions involving *lig4∆* was also apparent in cells treated with HU, although for unknown reasons *lig4∆* did enhance UV sensitivity in the *brc1∆ mus81∆* background (Fig 2B).

### Increased Rad52 foci in *brc1∆ pku80∆* cells

**Fig 4:**
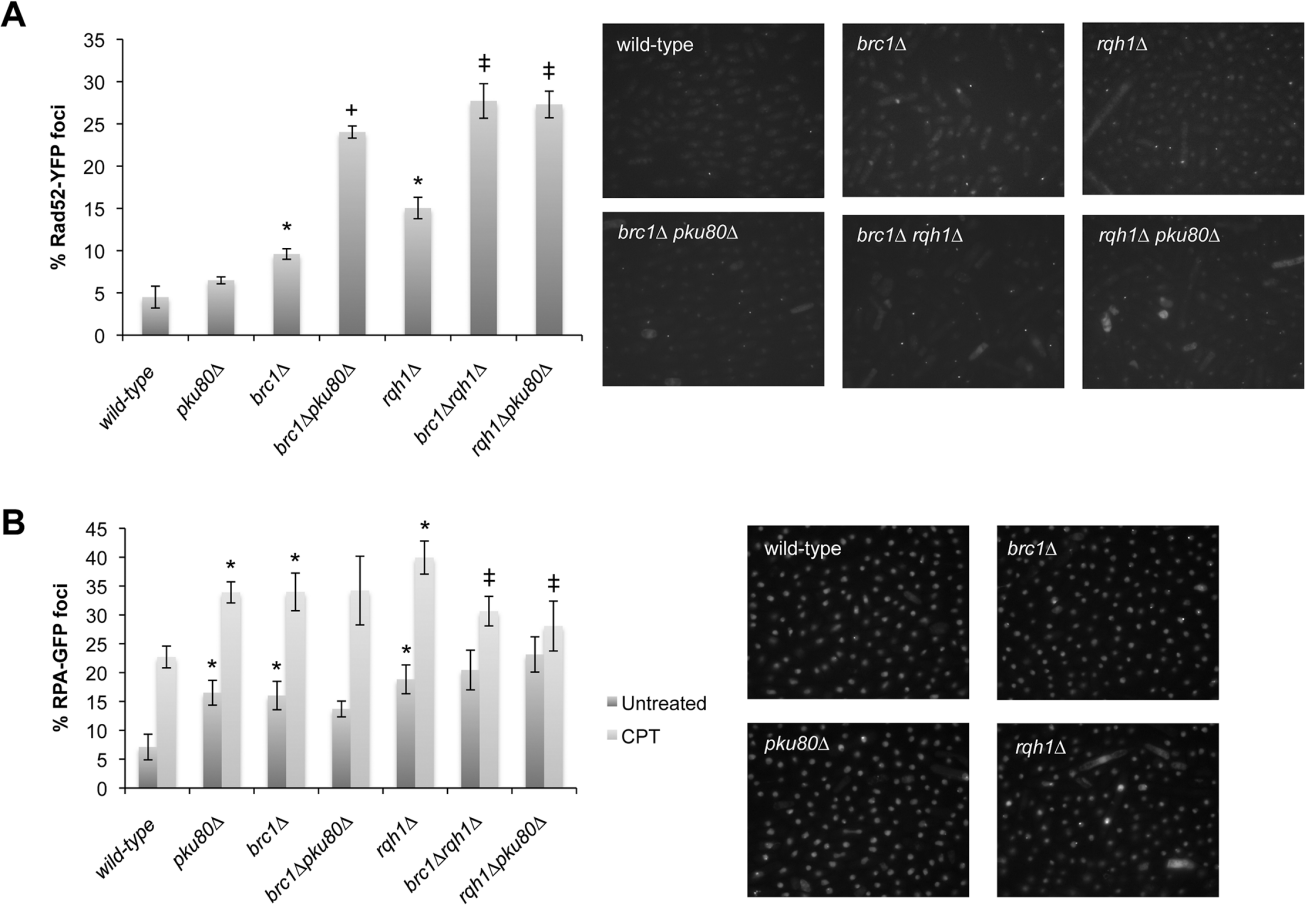
Increased Rad52 foci in *brc1∆ pku80∆* and *rqh1∆ pku80∆* cells. Cells expressing Rad52-YFP (panel A) or Rad11(RPA)-GFP (panel B) were cultured in minimal medium at 25°C until mid-log phase. Foci were scored in three independent experiments. Error bars correspond to standard deviations of the means. Asterisk depicts statistically significant differences with wild type, + symbol with *brc1∆*, and ‡ with *rqh1∆*, as determined by two-tailed Student T-test, p-value ≤ 0.05.

Rad52 (aka Rad22 in fission yeast) is essential for HR repair and many mutants with genome maintenance defects have increased numbers of Rad52 nuclear foci [35–37]. As an independent test of genome instability in *brc1∆ pku80∆* cells, we monitored Rad52-YFP foci in this strain and its parents. The incidence of Rad52 foci was modestly increased in *brc1∆* cells (9%) and *pku80∆* (6.5%) cells compared to wild type (4%). There was a synergistic increase of Rad52 foci in *brc1∆ pku80∆* cells, with 24% of these cells having at least one Rad52 focus (Fig 4A). This result supported the idea that the SSL interaction between *brc1∆* and *pku80∆* is caused by increased replication fork instability.

We also used this assay to assess the SSL interaction between *rqh1∆* and *pku80∆* [29]. We found that the *rqh1∆* mutation caused a ~3-fold increase in cells with Rad52 foci (15%), indicating that replication fork instability increases in *rqh1∆* cells (Fig 4A). There was a further increase in Rad52 foci in *rqh1∆ pku80∆* cells to 25%. A comparable value was obtained in *brc1∆ rqh1∆* cells, which comports with the observation that these cells and *rqh1∆ pku80∆* cells reveal strong SSL interactions.

The same assay was also performed with RPA, which is the major single-stranded DNA binding factor in fission yeast (Fig 4B). All of the single mutants had an increased percentage of cells with RPA foci (16–18%) as compared to wild type (7.5%), but there was little or no increase in the double mutant combinations (14–23%). All of strains increased RPA foci when treated with CPT. Taken together these data strongly suggest that Ku has an important role in protecting genome integrity in the absence of Brc1 or Rqh1.

### Exo1 contributes to cell survival in *brc1∆ pku80∆* cells

Ku and the Mre11-Rad50-Nbs1 (MRN) protein complex rapidly associate with DSBs *in vivo* [38, 39]. In the presence of the MRN cofactor Ctp1, which is orthologous to CtIP in mammals and Sae2 in budding yeast, the MRN endonuclease complex initiates 5’-to-3’ resection of the DSB and this process dislodges Ku [24, 40]. Exo1 exonuclease subsequently binds the 3’ single-strand tail and catalyzes further resection. In the absence of Ctp1 or even the entire MRN complex, Ku blocks the resection activity of Exo1 exonuclease. This DNA end blocking activity of Ku is independent of Ligase IV and other NHEJ factors [39]. We wondered whether this anti-Exo1 activity might explain the requirement for Ku in *brc1∆* cells. In support of this possibility, studies in *S*. *cerevisiae* showed that elimination of Exo1 stabilizes replication forks and reduces genotoxin sensitivity in cells lacking the checkpoint kinase Rad53, which is orthologous to human Chk2 and fission yeast Cds1. Other studies have also suggested that Exo1 might process reversed replication forks, which depending on the circumstances might enhance or impair cell survival [41, 42].

**Fig 5:**
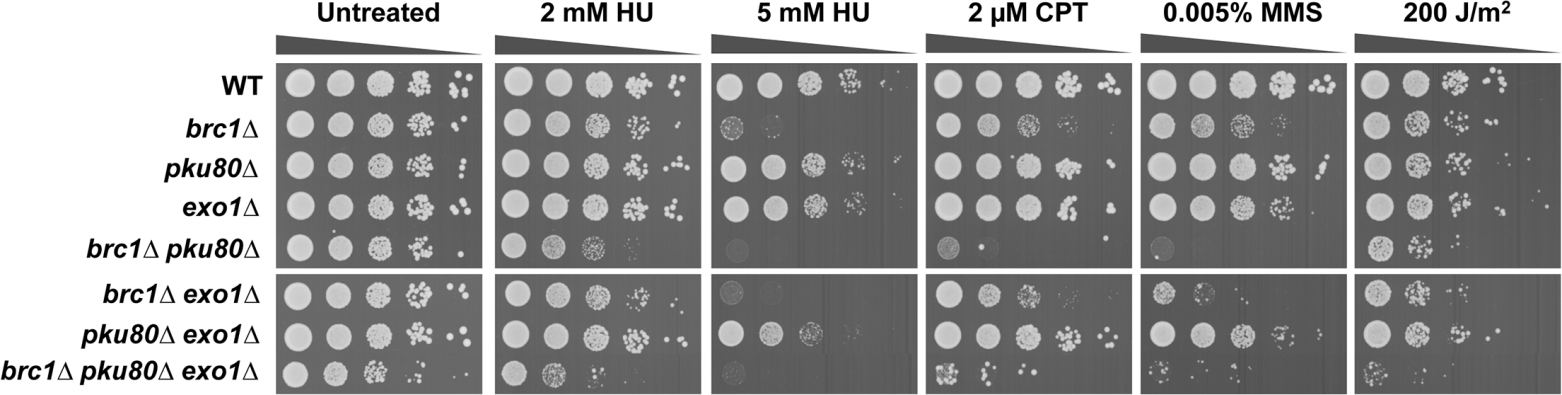
Elimination of Exo1 enhances genotoxin sensitivity in *brc1∆ pku80∆* cells. Tenfold serial dilutions of cells were exposed to the indicated DNA-damaging agents, and plates were incubated at 30°C for 3 to 4 days.

To explore whether Ku has an important Exo1-blocking activity in *brc1∆* cells we analyzed the genetic interactions of *brc1*, *pku80* and *exo1*. Deletion of Exo1 did not impair growth although it did appear to modestly increase sensitivity to HU and MMS, suggesting it plays a positive role in survival of replication stress (Fig 5). Deletion of Exo1 did not appear to impair or enhance growth in untreated *brc1∆* or *pku80∆* cells, although it did modestly increase MMS sensitivity in *brc1∆* cells. Most strikingly, elimination of Exo1 in *brc1∆ pku80∆* cells clearly impaired growth in the absence of genotoxins (Fig 5, untreated). These data argue against the anti-Exo1 activity of Ku playing a positive role in *brc1∆* cells; indeed, Exo1 contributes to cell survival in the *brc1∆ pku80∆* background.

### Ku localizes near the RFP4 replication fork barrier in the rDNA

**Fig 6:**
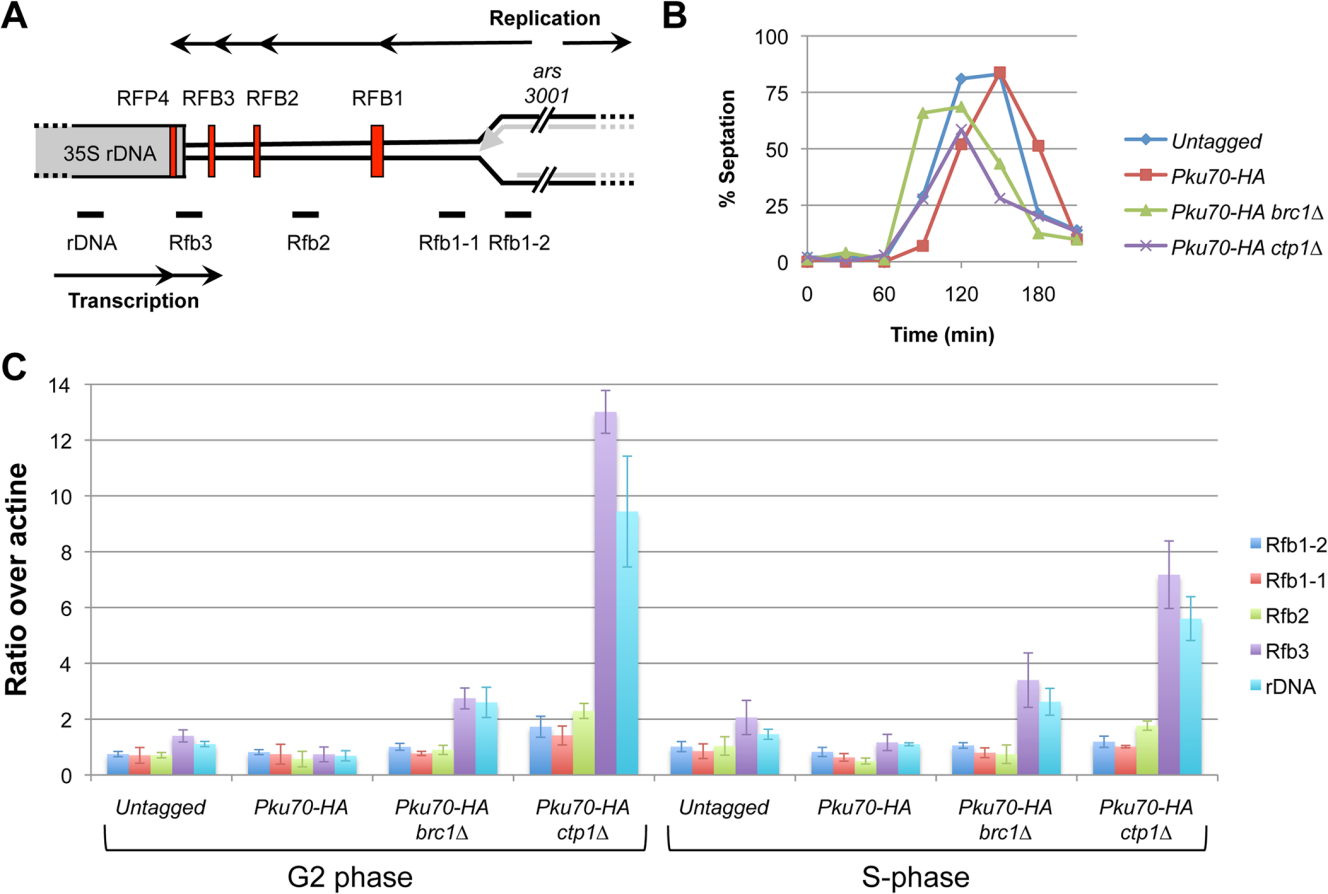
Enrichment of Pku70 at the rDNA RFP4 replication fork barrier in *brc1∆* and *ctp1∆* mutants. (A) Diagram of a single rDNA repeat (not to scale) shows the location of the four replication fork barriers (red vertical bars) relative to the 35S rDNA genes, the direction of replication (upper black arrow) from the *ars3001* replication origin, the direction of 35S rDNA transcription (lower black arrow) and qPCR primer locations, below graph. (B) Cells were synchronized in G2-phase using the *cdc25-22* allele and S-phase progression was monitored using septation index. (C) Pku70 ChIP at the rDNA was performed in untagged, wild type, *brc1∆*, and *ctp1∆* strains synchronized by *cdc25-22* block and analyzed by qPCR with the indicated primers. The G2-phase and S-phase samples correspond to 0 and 60 minutes respectively in this experiment.

The synergistic growth defect of *brc1∆ pku80∆* cells, their hypersensitivity to genotoxins that disturb DNA replication, and the critical requirement for Mus81 in these cells, all strongly suggested that Ku plays an important role in stabilizing replication forks in *brc1∆* cells. We therefore investigated whether Ku co-localizes with stalled forks. For these studies we focused on the replication fork barriers (RFBs) in the ribosomal DNA (rDNA) loci because we had previously found that the majority of spontaneous Brc1 foci co-localize with the nucleolus, which contains the rDNA occurring as tandem repeats in the subtelomeric arms of chromosome 3 [8, 43, 44]. A diagram of a single rDNA repeat is shown in Fig 6A. Each rDNA repeat consists of the 35S rDNA genes, a replication origin (*ars3001*), and four distinct replication fork barriers (RFB1-3 and RFP4). The rDNA genes are particularly vulnerable to recombination triggered by replication fork stalling or collapse because the repetitive sequences provide good substrates for homology-directed repair [45].

We carried out chromatin immunoprecipitation (ChIP) to measure Pku70 enrichment throughout the rDNA locus using the indicated primers (Fig 6A). We performed these ChIP studies on cells enriched in S-phase by using the *cdc25-22* arrest and release protocol [6]. We did not detect enrichment of Pku70 at any of the rDNA sites in the wild type *brc1* ^+^ strain. However, ChIP revealed ~2-fold enrichment of Pku70 in *brc1∆* cells specifically using the RFP4 and rDNA primer sets (Fig 6C). These primers flank the RFP4 barrier. Fork pausing at RFP4 is caused by collisions between the transcription and replication machineries, whereas replication pausing at RFB1-3 is programmed and depends on the Swi1-Swi3 complex [44]. Unexpectedly, Pku70 enrichment near RFP4 was approximately equal in the G2 and S phase samples, suggesting delayed proteolytic removal of Ku topologically trapped on double-stranded DNA either by HR repair or formation of extrachromosomal ribosomal DNA circles [46].

As mentioned above, we had previously found that the MRN protein complex and Ctp1 are required to displace Ku from DSBs through endonuclease processing of the DNA end [24]. To investigate whether this process applies in the RFP4 region, we used ChIP to monitor Pku70 localization in a *ctp1∆* strain. Interestingly, in both the G2 and S phase samples we again found Pku70 enrichment using primers that flanked RFP4 (Fig 6C). The overall pattern was similar to that observed in *brc1∆* cells except there was greater enrichment in *ctp1∆* cells. These findings suggest that Ku localizes to DNA ends formed regressed or broken replication forks at RFP4, and the MRN protein complex and Ctp1 displace Ku from these DNA ends by the same mechanism that occurs at DSBs formed by clastogens or DNA endonucleases.

### Increased requirement for Brc1 in *swi1∆* and *swi3∆* cells

**Fig 7:**
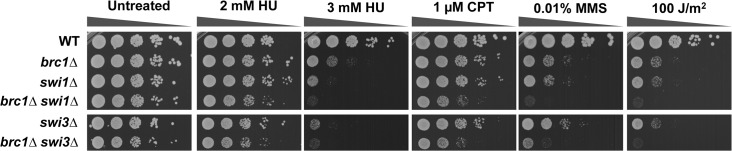
Requirement for Brc1 is enhanced in *swi1∆* and *swi3∆* mutants. Tenfold serial dilutions of cells were exposed to the indicated DNA-damaging agents, and plates were incubated at 30°C for 3 to 4 days.

The enrichment of Ku specifically near the RFP4 pause site in *brc1∆* cells suggests that Brc1 may be important for stabilizing forks in regions of replisome-transcriptosome collisions. Previous studies revealed that elimination of RFB1-3 fork pause sites in *swi1∆* and *swi3∆* mutants dramatically increases fork pausing at RFP4, likely resulting from increased replisome-transcriptosome collisions [44]. This effect correlates with increased Rad52 foci and a critical requirement for Mus81 and other HR enzymes in *swi1∆* and *swi3∆* mutants [35, 47]. If Brc1 is important for stabilizing replication forks at sites of replisome-transcriptosome collisions, as suggested by our results and recent studies [16, 48], we would expect a significant SSL interaction between *brc1∆* and *swi1∆* or *swi3∆*. Indeed, we detected an interaction with *swi3∆* in our *brc1∆* E-MAP screen, with the *brc1∆ swi3∆* mutant ranking 26^th^ with an E-MAP score of -4.6. This interaction was confirmed by construction of a *brc1∆ swi3∆* strain by tetrad analysis (Fig 7). This negative genetic interaction was enhanced in the presence of genotoxins. Construction of a *brc1∆ swi1∆* yielded an obvious SSL interaction only in the presence of genotoxins (Fig 7). The stronger SSL interaction with *swi3∆* is consistent with earlier studies showing that *swi3∆* mutants are more sensitive to replicative stress [35, 47].

## Discussion

Here, we have described how Ku contributes to cell survival in the absence of Brc1. This genetic interaction suggested that *brc1∆* cells suffer increased DSBs that require NHEJ for repair, but we found that Ligase IV was not required in *brc1∆* cells. As Ligase IV is essential for NHEJ, we concluded that the requirement for Ku in *brc1∆* cells does not involve NHEJ. Instead, we propose that Ku protects DNA ends that arise at stalled replication forks in *brc1∆* cells.

Our data indicates that replicative stress from endogenous sources is toxic in *brc1∆ pku80∆* cells. Rad3 (ATR) creates γH2A at key genomic features during S-phase, including natural replication fork barriers, retrotransposons, heterochromatin domains and rDNA repeats [6]. The rDNA repeats have multiple fork barriers and a region of frequent collisions between DNA and RNA polymerases; indeed, we found that most spontaneous Brc1 nuclear foci localize in the nucleolus with the rDNA repeats [8]. Brc1 is also enriched in pericentromeric heterochromatin, which is another chromosomal domain of frequent polymerase collisions [16, 49].

The *brc1∆ pku80∆* SSL interaction is enhanced when cells are treated with genotoxins that cause replicative stress. These genetic interactions are impressive because *pku80∆* cells are largely insensitive to replication stress. Indeed, NHEJ should be useless as a repair mechanism for collapsed replication forks. This supposition is consistent with the absence SSL interactions between *brc1∆* and *lig4∆* mutations. Indeed, NHEJ defective strains are not generally sensitive to clastogens because G1 phase is very brief except during nutrient limitation [20, 50]. These considerations make the SSL interaction between *brc1∆* and *pku80∆* all the more striking.

The significantly higher percentage of cells with Rad52 foci in *brc1∆ pku80∆* cells suggests that DNA replication-associated lesions are increased or inefficiently repaired. The relatively modest increase of Rad52 foci in *brc1∆* cells might involve activation of dormant origins, which could explain the epistatic genetic interactions involving Brc1 and Mus81 [9, 15]. As Rad52 is essential for all HR repair, the increased Rad52 foci in *brc1∆ pku80∆* cells might indicate increased replication fork breakage. However, Rad52 might associate with ssDNA at stalled, regressed, rearranged, terminated or collapsed replication forks. These structures might be irreparable or repaired without HR. In either case, Ku’s DNA end-binding specificity suggests that broken or regressed forks are connected to the large increase in Rad52 foci in *brc1∆ pku80∆* cells.

Mus81-Eme1 is the only Holliday junction resolvase in fission yeast and is therefore essential for repairing broken replication forks [19]. The poor growth of *mus81∆* mutants is not exacerbated by *brc1∆* or *pku80∆* mutations, indicating that neither mutation increases fork collapse or breakage. However, the very poor growth of *mus81∆ brc1∆ pku80∆* cells indicates that Ku prevents replication fork collapse, rearrangement or breakage in the absence of Brc1. This result indicates that many of the Rad52 foci in *brc1∆ pku80∆* cells indicate HR repair of replication forks.

Mre11 complex and Sae2/Ctp1 are required to remove Ku from DNA ends [38, 39]. Elimination of Ku improves HR repair in mutants lacking Mre11 endonuclease activity or Sae2/Ctp1, but this suppression requires Exo1, suggesting that Ku blocks Exo1-mediated resection of DSBs. However, the Exo1-blocking activity of Ku does not explain the *brc1∆ pku80∆* SSL interaction because we found that that *exo1∆* exacerbates the *brc1∆ pku80∆* growth defect. The *brc1∆ exo1∆* strain did not have an obvious growth defect, suggesting that elimination of Exo1 does not increase replicative stress. Instead, we suspect that the DNA end resection activity of Exo1 facilitates repair of broken forks in *brc1∆ pku80∆* cells.

Taken together, these data suggest that Ku prevents replication fork collapse or rearrangement in the absence of Brc1. The increased association of Ku at RFB4 but not RFB1 in *brc1∆* cells suggests that fork pausing caused by transcriptosome-replisome collisions creates a requirement for Ku in *brc1∆* cells. This model is consistent with SSL interactions of *brc1∆* with *swi1∆* and *swi3∆* mutations, which increase transcriptosome-replisome collisions at RFB4.

Our findings contribute to a growing body of evidence that Ku has an under appreciated role in responding to replication stress. Of special note are the studies of Ishikawa and colleagues, who described a critical non-NHEJ requirement for Ku in *rqh1∆* cells exposed to replication stress [29]. Extending their findings, we found that eliminating Ku increases Rad52 foci in *rqh1∆* cells in the absence of replication stress genotoxins. This non-canonical role of Ku in S-phase might be conserved because Ku improves replication stress survival in budding yeast mutants lacking Sgs1, which is the ortholog of Rqh1 [51, 52]. Furthermore, studies with mammalian cells have implicated NHEJ factors such as DNA-PKcs and Artemis in recovery from replication stress [53].

**Fig 8:**
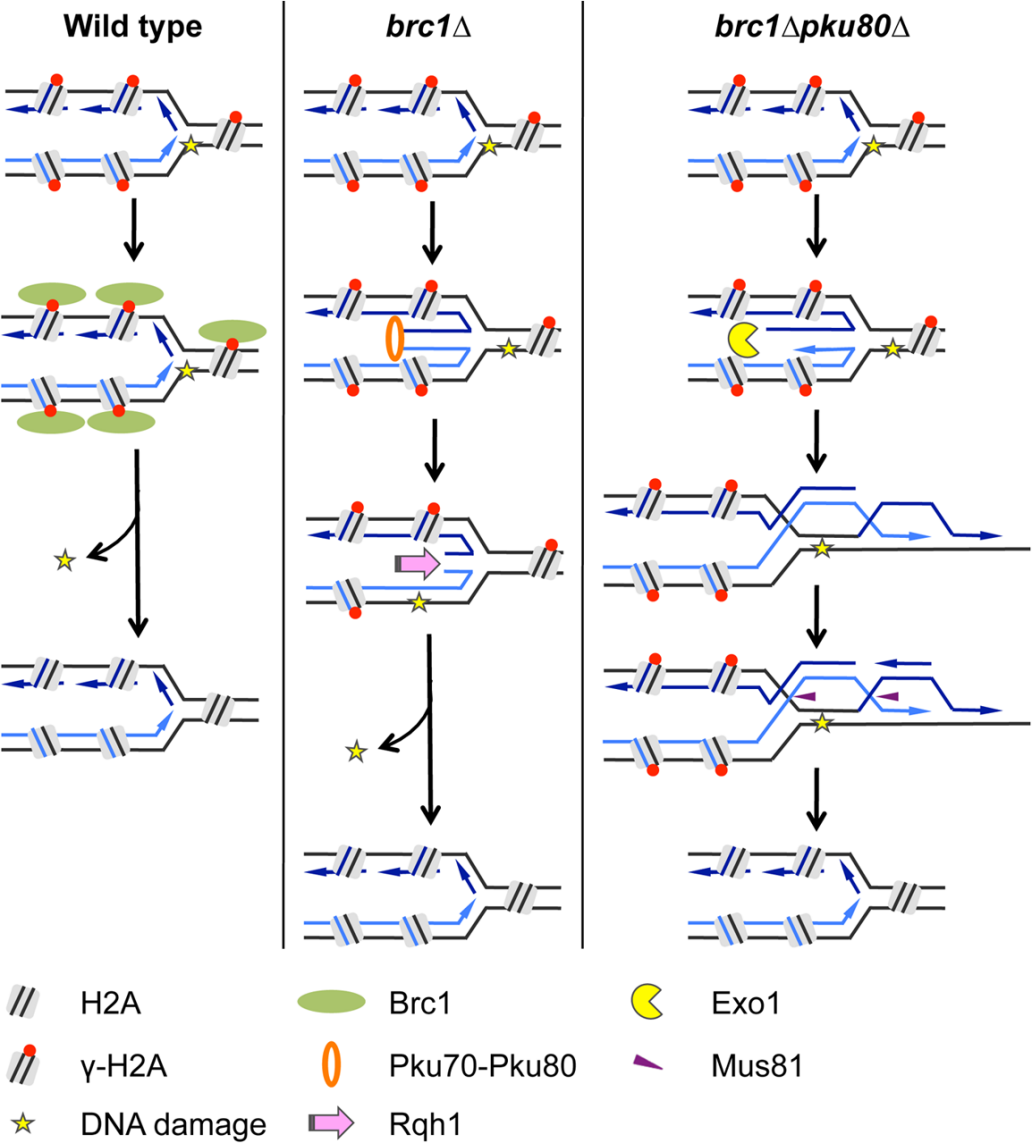
Model proposed to explain the requirement for Ku in *brc1∆* cells. In wild type cells, replication fork stalling leads to phosphorylation of histone H2A followed by recruitment of Brc1, which stabilizes the replication fork. In *brc1∆* cells, stalled replication forks are prone to fork reversal. Ku binds the exposed DNA end of the chicken foot structure to prevent fork collapse or rearrangement. This activity of Ku favors resetting of replication fork which increases the likelihood of successful completion of DNA replication. In *brc1∆ pku80∆* cells, stalled replication forks undergo homologous recombination without DSB formation as shown or collapse (possibly through a Mus81-dependent mechanism) and reform through homologous recombination (not shown). In either case Mus81-Eme1 is required to resolve Holliday junction-like structures and Exo1 contributes to resection necessary for HR.

In Fig 8 we propose a model that we believe most economically reconciles our findings with the known biochemical properties of the involved proteins, particularly Ku’s high affinity for double-stranded DNA ends. In this model Brc1 stabilizes replication forks to prevent their regression. Increased fork regression in the absence of Brc1 generates a chicken foot structure containing a DNA end that is bound by Ku. By binding nascent chicken feet, Ku stabilizes the replication fork and reduces the probability that it will break or undergo homologous recombination to form Holliday junctions that require resolution by Mus81-Eme1. Our future experiments will be aimed at testing this model.

## Supporting Information

S1 Table *S*. *pombe* strains used in this study. (DOCX)

## References

[1] Zeman MK, Cimprich KA. Causes and consequences of replication stress. Nature cell biology. 2014;16(1):2–9. 10.1038/ncb2897 . 24366029 PMC4354890

[2] Lambert S, Carr AM. Impediments to replication fork movement: stabilisation, reactivation and genome instability. Chromosoma. 2013;122(1–2):33–45. 10.1007/s00412-013-0398-9 . 23446515

[3] Hills SA, Diffley JF. DNA replication and oncogene-induced replicative stress. Current biology: CB. 2014;24(10):R435–44. 10.1016/j.cub.2014.04.012 . 24845676

[4] Branzei D, Foiani M. Maintaining genome stability at the replication fork. Nat Rev Mol Cell Biol. 2010;11(3):208–19. 10.1038/nrm2852 . 20177396

[5] Bonner WM, Redon CE, Dickey JS, Nakamura AJ, Sedelnikova OA, Solier S, et al GammaH2AX and cancer. Nature reviews Cancer. 2008;8(12):957–67. 10.1038/nrc2523 . 19005492 PMC3094856

[6] Rozenzhak S, Mejia-Ramirez E, Williams JS, Schaffer L, Hammond JA, Head SR, et al Rad3 decorates critical chromosomal domains with gammaH2A to protect genome integrity during S-Phase in fission yeast. PLoS Genet. 2010;6(7):e1001032 10.1371/journal.pgen.1001032 . 20661445 PMC2908685

[7] Nakamura TM, Du LL, Redon C, Russell P. Histone H2A phosphorylation controls Crb2 recruitment at DNA breaks, maintains checkpoint arrest, and influences DNA repair in fission yeast. Mol Cell Biol. 2004;24(14):6215–30. 10.1128/MCB.24.14.6215-6230.2004 . 15226425 PMC434244

[8] Williams JS, Williams RS, Dovey CL, Guenther G, Tainer JA, Russell P. gammaH2A binds Brc1 to maintain genome integrity during S-phase. EMBO J. 2010;29(6):1136–48. Epub 2010/01/23. 10.1038/emboj.2009.413 . 20094029 PMC2845269

[9] Sheedy DM, Dimitrova D, Rankin JK, Bass KL, Lee KM, Tapia-Alveal C, et al Brc1-mediated DNA repair and damage tolerance. Genetics. 2005;171(2):457–68. Epub 2005/06/24. 10.1534/genetics.105.044966 . 15972456 PMC1456763

[10] Wehrkamp-Richter S, Hyppa RW, Prudden J, Smith GR, Boddy MN. Meiotic DNA joint molecule resolution depends on Nse5-Nse6 of the Smc5-Smc6 holocomplex. Nucleic acids research. 2012;40(19):9633–46. 10.1093/nar/gks713 . 22855558 PMC3479181

[11] Verkade HM, Bugg SJ, Lindsay HD, Carr AM, O'Connell MJ. Rad18 is required for DNA repair and checkpoint responses in fission yeast. Mol Biol Cell. 1999;10(9):2905–18. . 10473635 10.1091/mbc.10.9.2905 PMC25529

[12] Pebernard S, Wohlschlegel J, McDonald WH, Yates JR 3rd, Boddy MN. The Nse5-Nse6 dimer mediates DNA repair roles of the Smc5-Smc6 complex. Mol Cell Biol. 2006;26(5):1617–30. 10.1128/MCB.26.5.1617-1630.2006 . 16478984 PMC1430260

[13] Pebernard S, McDonald WH, Pavlova Y, Yates JR 3rd, Boddy MN. Nse1, Nse2, and a novel subunit of the Smc5-Smc6 complex, Nse3, play a crucial role in meiosis. Mol Biol Cell. 2004;15(11):4866–76. 10.1091/mbc.E04-05-0436 . 15331764 PMC524734

[14] Morikawa H, Morishita T, Kawane S, Iwasaki H, Carr AM, Shinagawa H. Rad62 protein functionally and physically associates with the smc5/smc6 protein complex and is required for chromosome integrity and recombination repair in fission yeast. Mol Cell Biol. 2004;24(21):9401–13. 10.1128/MCB.24.21.9401-9413.2004 . 15485909 PMC522231

[15] Bass KL, Murray JM, O'Connell MJ. Brc1-dependent recovery from replication stress. J Cell Sci. 2012;125(Pt 11):2753–64. 10.1242/jcs.103119 . 22366461 PMC3403237

[16] Lee SY, Rozenzhak S, Russell P. gammaH2A-binding protein Brc1 affects centromere function in fission yeast. Mol Cell Biol. 2013;33(7):1410–6. 10.1128/MCB.01654-12 . 23358415 PMC3624265

[17] McGlynn P, Lloyd RG. Recombinational repair and restart of damaged replication forks. Nat Rev Mol Cell Biol. 2002;3(11):859–70. 10.1038/nrm951 . 12415303

[18] Costes A, Lambert SA. Homologous recombination as a replication fork escort: fork-protection and recovery. Biomolecules. 2012;3(1):39–71. 10.3390/biom3010039 . 24970156 PMC4030885

[19] Roseaulin L, Yamada Y, Tsutsui Y, Russell P, Iwasaki H, Arcangioli B. Mus81 is essential for sister chromatid recombination at broken replication forks. EMBO J. 2008;27(9):1378–87. 10.1038/emboj.2008.65 . 18388861 PMC2374842

[20] Forsburg SL, Rhind N. Basic methods for fission yeast. Yeast. 2006;23(3):173–83. 10.1002/yea.1347 . 16498704 PMC5074380

[21] Bahler J, Wu JQ, Longtine MS, Shah NG, McKenzie A 3rd, Steever AB, et al Heterologous modules for efficient and versatile PCR-based gene targeting in Schizosaccharomyces pombe. Yeast. 1998;14(10):943–51. 10.1002/(SICI)1097-0061(199807)14:10<943::AID-YEA292>3.0.CO;2-Y . 9717240

[22] Roguev A, Bandyopadhyay S, Zofall M, Zhang K, Fischer T, Collins SR, et al Conservation and rewiring of functional modules revealed by an epistasis map in fission yeast. Science. 2008;322(5900):405–10. 10.1126/science.1162609 18818364 PMC2753251

[23] Nakamura TM, Moser BA, Russell P. Telomere binding of checkpoint sensor and DNA repair proteins contributes to maintenance of functional fission yeast telomeres. Genetics. 2002;161(4):1437–52. . 12196391 10.1093/genetics/161.4.1437 PMC1462227

[24] Langerak P, Mejia-Ramirez E, Limbo O, Russell P. Release of Ku and MRN from DNA ends by Mre11 nuclease activity and Ctp1 is required for homologous recombination repair of double-strand breaks. PLoS Genet. 2011;7(9):e1002271 Epub 2011/09/21. 10.1371/journal.pgen.1002271 . 21931565 PMC3169521

[25] Sanchez A, Sharma S, Rozenzhak S, Roguev A, Krogan NJ, Chabes A, et al Replication fork collapse and genome instability in a deoxycytidylate deaminase mutant. Mol Cell Biol. 2012;32(21):4445–54. 10.1128/MCB.01062-12 . 22927644 PMC3486156

[26] Roguev A, Wiren M, Weissman JS, Krogan NJ. High-throughput genetic interaction mapping in the fission yeast Schizosaccharomyces pombe. Nat Methods. 2007;4(10):861–6. 10.1038/nmeth1098 . 17893680

[27] Kim DU, Hayles J, Kim D, Wood V, Park HO, Won M, et al Analysis of a genome-wide set of gene deletions in the fission yeast Schizosaccharomyces pombe. Nat Biotechnol. 2010;28(6):617–23. 10.1038/nbt.1628 . 20473289 PMC3962850

[28] Lieber MR. The mechanism of double-strand DNA break repair by the nonhomologous DNA end-joining pathway. Annual review of biochemistry. 2010;79:181–211. 10.1146/annurev.biochem.052308.093131 . 20192759 PMC3079308

[29] Miyoshi T, Kanoh J, Ishikawa F. Fission yeast Ku protein is required for recovery from DNA replication stress. Genes Cells. 2009;14(9):1091–103. 10.1111/j.1365-2443.2009.01337.x . 19682091

[30] Baumann P, Cech TR. Protection of telomeres by the Ku protein in fission yeast. Mol Biol Cell. 2000;11(10):3265–75. . 11029034 10.1091/mbc.11.10.3265 PMC14990

[31] Kibe T, Tomita K, Matsuura A, Izawa D, Kodaira T, Ushimaru T, et al Fission yeast Rhp51 is required for the maintenance of telomere structure in the absence of the Ku heterodimer. Nucleic acids research. 2003;31(17):5054–63. . 12930956 10.1093/nar/gkg718 PMC212814

[32] Doe CL, Ahn JS, Dixon J, Whitby MC. Mus81-Eme1 and Rqh1 involvement in processing stalled and collapsed replication forks. The Journal of biological chemistry. 2002;277(36):32753–9. 10.1074/jbc.M202120200 . 12084712

[33] Boddy MN, Lopez-Girona A, Shanahan P, Interthal H, Heyer WD, Russell P. Damage tolerance protein Mus81 associates with the FHA1 domain of checkpoint kinase Cds1. Mol Cell Biol. 2000;20(23):8758–66. . 11073977 10.1128/mcb.20.23.8758-8766.2000 PMC86503

[34] Boddy MN, Gaillard PH, McDonald WH, Shanahan P, Yates JR 3rd, Russell P. Mus81-Eme1 are essential components of a Holliday junction resolvase. Cell. 2001;107(4):537–48. doi: S0092-8674(01)00536-0 [pii]. . 11719193 10.1016/s0092-8674(01)00536-0

[35] Noguchi E, Noguchi C, Du LL, Russell P. Swi1 prevents replication fork collapse and controls checkpoint kinase Cds1. Mol Cell Biol. 2003;23(21):7861–74. . 14560029 10.1128/MCB.23.21.7861-7874.2003 PMC207622

[36] Meister P, Poidevin M, Francesconi S, Tratner I, Zarzov P, Baldacci G. Nuclear factories for signalling and repairing DNA double strand breaks in living fission yeast. Nucleic acids research. 2003;31(17):5064–73. . 12930957 10.1093/nar/gkg719 PMC212815

[37] Du LL, Nakamura TM, Moser BA, Russell P. Retention but not recruitment of Crb2 at double-strand breaks requires Rad1 and Rad3 complexes. Mol Cell Biol. 2003;23(17):6150–8. . 12917337 10.1128/MCB.23.17.6150-6158.2003 PMC180945

[38] Mimitou EP, Symington LS. DNA end resection—unraveling the tail. DNA repair. 2011;10(3):344–8. 10.1016/j.dnarep.2010.12.004 . 21227759 PMC3046306

[39] Langerak P, Russell P. Regulatory networks integrating cell cycle control with DNA damage checkpoints and double-strand break repair. Philosophical transactions of the Royal Society of London Series B, Biological sciences. 2011;366(1584):3562–71. 10.1098/rstb.2011.0070 . 22084383 PMC3203453

[40] Limbo O, Chahwan C, Yamada Y, de Bruin RA, Wittenberg C, Russell P. Ctp1 is a cell-cycle-regulated protein that functions with Mre11 complex to control double-strand break repair by homologous recombination. Mol Cell. 2007;28(1):134–46. doi: S1097-2765(07)00621-1 [pii] 10.1016/j.molcel.2007.09.009 . 17936710 PMC2066204

[41] Segurado M, Diffley JF. Separate roles for the DNA damage checkpoint protein kinases in stabilizing DNA replication forks. Genes Dev. 2008;22(13):1816–27. doi: 22/13/1816 [pii] 10.1101/gad.477208 . 18593882 PMC2492668

[42] Cotta-Ramusino C, Fachinetti D, Lucca C, Doksani Y, Lopes M, Sogo J, et al Exo1 processes stalled replication forks and counteracts fork reversal in checkpoint-defective cells. Mol Cell. 2005;17(1):153–9. 10.1016/j.molcel.2004.11.032 . 15629726

[43] Sanchez-Gorostiaga A, Lopez-Estrano C, Krimer DB, Schvartzman JB, Hernandez P. Transcription termination factor reb1p causes two replication fork barriers at its cognate sites in fission yeast ribosomal DNA in vivo. Mol Cell Biol. 2004;24(1):398–406. . 14673172 10.1128/MCB.24.1.398-406.2004 PMC303360

[44] Krings G, Bastia D. swi1- and swi3-dependent and independent replication fork arrest at the ribosomal DNA of Schizosaccharomyces pombe. Proc Natl Acad Sci U S A. 2004;101(39):14085–90. 10.1073/pnas.0406037101 . 15371597 PMC521093

[45] Tsang E, Carr AM. Replication fork arrest, recombination and the maintenance of ribosomal DNA stability. DNA repair. 2008;7(10):1613–23. 10.1016/j.dnarep.2008.06.010 . 18638573

[46] Postow L. Destroying the ring: Freeing DNA from Ku with ubiquitin. FEBS letters. 2011;585(18):2876–82. 10.1016/j.febslet.2011.05.046 . 21640108 PMC3172340

[47] Noguchi E, Noguchi C, McDonald WH, Yates JR 3rd, Russell P. Swi1 and Swi3 are components of a replication fork protection complex in fission yeast. Mol Cell Biol. 2004;24(19):8342–55. 10.1128/MCB.24.19.8342-8355.2004 . 15367656 PMC516732

[48] Lee SY, Russell P. Brc1 links replication stress response and centromere function. Cell Cycle. 2013;12(11):1665–71. 10.4161/cc.24900 . 23656778 PMC3713124

[49] Zaratiegui M, Castel SE, Irvine DV, Kloc A, Ren J, Li F, et al RNAi promotes heterochromatic silencing through replication-coupled release of RNA Pol II. Nature. 2011;479(7371):135–8. 10.1038/nature10501 . 22002604 PMC3391703

[50] Ferreira MG, Cooper JP. Two modes of DNA double-strand break repair are reciprocally regulated through the fission yeast cell cycle. Genes Dev. 2004;18(18):2249–54. 10.1101/gad.315804 . 15371339 PMC517518

[51] Ui A, Seki M, Ogiwara H, Onodera R, Fukushige S, Onoda F, et al The ability of Sgs1 to interact with DNA topoisomerase III is essential for damage-induced recombination. DNA repair. 2005;4(2):191–201. 10.1016/j.dnarep.2004.09.002 . 15590327

[52] Yamana Y, Maeda T, Ohba H, Usui T, Ogawa HI, Kusano K. Regulation of homologous integration in yeast by the DNA repair proteins Ku70 and RecQ. Mol Genet Genomics. 2005;273(2):167–76. 10.1007/s00438-005-1108-y . 15803320

[53] Allen C, Ashley AK, Hromas R, Nickoloff JA. More forks on the road to replication stress recovery. J Mol Cell Biol. 2011;3(1):4–12. 10.1093/jmcb/mjq049 . 21278446 PMC3030971

