# Supplementary material for: Ku Stabilizes Replication Forks in the Absence of Brc1
Source: PLoS One. 2015 May 12;10(5):e0126598. doi: 10.1371/journal.pone.0126598 (PMC4428774; doi:10.1371/journal.pone.0126598)
Supplement: S1 Table — (DOCX) [file pone.0126598.s001.docx]

**Supporting information**

**S1 Table.** ***S. pombe* strains used in this study.**

| **Strain** | **Genotype** | **Source** |
| --- | --- | --- |
| AS5121 | *h- leu1-32* | Lab stock |
| AS127 | *h- leu1-32 ade6M210/216* | Lab stock |
| AS5122 | *h- leu1-32 brc1::hphMX6* | Lab stock |
| AS1570 | *h- leu1-32 pku70::KanMX4* | This study |
| SC4082 | *h^+^ leu1-32 ura4-D18 pku80::kanMX6* | Lab stock |
| AS156 | *h^+^ leu1-32 pku80::KanMX6* | Lab stock |
| AS1215 | *h^90^ leu1-32 pku80::KanMX6* | This study |
| AS5148 | *h^+^ leu1-32 mus81::NatMX6* | Lab stock |
| AS627 | *h^+^ leu1-32 his3-D1 lig4::KanMX4* | Lab stock |
| OL4175 | *h- leu1-32 ura4-D18 exo1::ura4* | Lab stock |
| AS1564 | *h- leu1-32 ura4-D18 brc1::HphMX6 pku70::KanMX4* | This study |
| AS152 | *h^+^ leu1-32 brc1::HphMX6 pku80::KanMX6* | This study |
| AS636 | *h^+^ leu1-32 brc1::HphMX6 mus81::NatMX6* | This study |
| AS604 | *h- leu1-32 ura4-D18 pku80::KanMX6 mus81::NatMX6* | This study |
| AS625 | *h^+^ leu1-32 ade6-210 his3-D1 brc1::HphMX6 lig4::KanMX4* | This study |
| AS656 | *h- leu1-32 ura4-D18 ade6-M210 lig4::KanMX4 mus81::NatMX6* | This study |
| AS381 | *h^+^ ura4-D18 leu1-32 brc1::HphMX6 exo1::ura4* | This study |
| AS379 | *h- ura4-D18 leu1-32 pku80::KanMX6 exo1::ura4* | This study |
| AS618 | *h^+^ leu1-32 pku80::KanMX6 brc1::HphMX6 mus81::NatMX6* | This study |
| AS658 | *h^+^* *leu1-32 ura4-D18 ade6-M210 his3-D lig4::KanMX4 brc1::HphMX6 mus81::NatMX6* | This study |
| AS405 | *h- ura4-D18 leu1-32 brc1::HphMX6 pku80::KanMX6 exo1::ura4* | This study |
| AS1250 | *h- leu1-32* | This study |
| AS1252 | *h^+^*  *leu1-32 brc1::HphMX6* | This study |
| AS1251 | *h^+^* *leu1-32 pku80::KanMX6* | This study |
| AS1253 | *h- leu1-32 brc1::HphMX6 pku80::KanMX6* | This study |
| AS1409 | *h^+^ leu1-32 ura4-D18 Rad22-YFP:kanMX6* | This study |
| AS1431 | *h^+^ leu1-32 ura4-D18 brc1::HphMX6 Rad22-YFP:kanMX6* | This study |
| AS1453 | *h- leu1-32 ura4-D18 pku80::KanMX6 Rad22-YFP:KanMX* | This study |
| AS1462 | *h^+^ leu1-32 ura4-D18 rqh1::ura4+ Rad22-YFP:KanMX* | This study |
| AS1460 | *h^+^ leu1-32 ura4-D18 brc1::HphMX6 pku80::KanMX6 Rad22-YFP:KanMX* | This study |
| AS1465 | *h^90^ leu1-32 ura4-D18 pku80::KanMX6 rqh1::ura4+Rad22-YFP:KanMX* | This study |
| AS1463 | *h^+^ leu1-32 ura4-D18 brc1::HphMX6 rqh1::ura4+ Rad22-YFP:KanMX* | This study |
| OL5102 | *h- leu1-32 ura4-D18 Rad11-GFP:HphMX6* | Lab stock |
| AS712 | *h- leu1-32 ura4-D18 brc1::HphMX6 Rad11-GFP:HphMX6* | This study |
| AS1427 | *h- leu1-32 ura4-D18 pku80::KanMX6 Rad11-GFP:HphMX6* | This study |
| AS1348 | *h- leu1-32 ura4-D18 rqh1::ura4 Rad11-GFP:HphMX6* | This study |
| AS1476 | *h- leu1-32 ura4-D18 pku80::KanMX6 brc1::HphMX6 Rad11-GFP:HphMX6* | This study |
| AS1474 | *h- leu1-32 ura4-D18 pku80::KanMX6 rqh1::ura4+ Rad11-GFP:HphMX6* | This study |
| AS1470 | *h- leu1-32 ura4-D18 brc1::HphMX6 rqh1::ura4+ Rad11-GFP:HphMX6* | This study |
| KS1483 | *h^+^ leu1-32 ura4-D18 cdc25-22* | Lab stock |
| AS1122 | *h^+^ leu1-32 ura4-D18 ade6-M210 his3-D1 cdc25-22 pku70-3HA:KanMX* | This study |
| AS1161 | *h^+^ leu1-32 ura4-D18 ade6-M210 his3-D1 cdc25-22 pku70-3HA:KanMX brc1::HphMX6* | This study |
| AS1151 | *h^+^ leu1-32 ura4-D18 ade6-M210 his3-D1 cdc25-22 pku70-3HA:KanMX ctp1::NatMX6* | This study |
| AS5138 | *h- leu1-32 swi1::KanMX* | Lab stock |
| AS644 | *h^+^ leu1-32 brc1::HphMX6 swi1::KanMX* | This study |
| AS647 | *h- leu1-32 swi3::KanMX6* | This study |
| AS650 | *h^+^ leu1-32 brc1::HphMX6 swi3::KanMX6* | This study |
